# The Association of Pre-stroke Psychosis and Post-stroke Levels of Health, Resource Utilization, and Care Process: A Register-Based Study

**DOI:** 10.3389/fneur.2018.01042

**Published:** 2018-12-03

**Authors:** Carl Willers, Katharina S. Sunnerhagen, Ingrid Lekander, Mia von Euler

**Affiliations:** ^1^Department of Clinical Science and Education, Karolinska Institutet, Stockholm, Sweden; ^2^Karolinska Institutet Stroke Research Network at Södersjukhuset, Solna, Sweden; ^3^Ivbar Institute AB, Stockholm, Sweden; ^4^Rehabilitation Medicine, Institute of Neuroscience and Physiology, The Sahlgenska Academy, University of Gothenburg, Gothenburg, Sweden; ^5^Medical Management Centre, Karolinska Institutet, Solna, Sweden; ^6^Department of Medicine, Karolinska Institutet, Solna, Sweden; ^7^Department of Clinical Pharmacology, Karolinska University Hospital, Solna, Sweden

**Keywords:** mental comorbidity, stroke, health equity, health outcomes, resource use

## Abstract

**Background:** While approximately one percent of the global population is formally diagnosed with psychosis or schizophrenia, the actual number is expected to be significantly higher. These patients often consume more healthcare resources and have poorer somatic health. In this study, we analyze potential differences in health, resources, and care process between stroke patients with and without a previous diagnosis of psychosis or schizophrenia.

**Methods:** Ischemic stroke patients from seven regions in Sweden were identified via ICD-10 codes (I63.0-9) in regional administrative systems and the Swedish Stroke Register, and approximately 70% of all ischemic stroke cases in Sweden during 2008–2011 were included (*n* = 46,350). Relevant patient-level data from national registries were linked to enable multivariate regression analysis, including data on socioeconomics, mortality, municipality services, and filled prescriptions. History of psychosis or schizophrenia was defined via ICD-10 codes F20-29 (*n* = 389).

**Results:** Patient-reported functional outcomes at 3 months and 1 year were significantly lower in the psychosis subgroup, and stroke recurrence was higher. Patients with pre-stroke psychosis did not receive the same levels of reperfusion treatment as the non-psychosis group. Time at the stroke unit was the same, as were first-year levels of somatic care, but dispensation of antihypertensives was less common.

**Conclusion:** Our findings emphasize the importance of taking mental comorbidity into account during stroke treatment as well as when evaluating indicators for health, resources, and the care process, since mental comorbidity such as psychosis or schizophrenia may have a significant impact the year preceding and the year succeeding the stroke event.

## Background

While approximately one percent of the global population is formally diagnosed with psychosis or schizophrenia, the actual number is expected to be significantly higher (1). The prevalence is equivalent in Sweden (1·04% with formal registered diagnosis in Stockholm, the largest administrative region) (2), a health system characterized by universal, tax-subsidized access to healthcare (3) and with need-based equal care as a formal aim within the health system (4). Individuals with a medical history of psychosis and/or schizophrenia generally consume more healthcare resources than patients without such a diagnosis (5), have generally worse somatic health including more cardiovascular risk factors, and are more likely to develop comorbidities (6–9). Circulatory disease is one of the major causes of excess death in people with psychiatric disorders, second to suicide (10, 11). This may be due to several factors, including lifestyle, poor identification and management of risk factors and the disease itself, lack of health literacy, and side effects of antipsychotic medication (8, 12), which may further increase the risk of cardiovascular events (13).

The way that individuals affected by pre-stroke psychosis or schizophrenia are treated and regain health after stroke is relatively unstudied. Psychosis patients have been shown to have poorer access to some procedures within cardiac and stroke care (14), also within a universal health system (15, 16), and individuals with pre-stroke psychosis have been shown to receive less stroke rehabilitation (17).

In this study, we analyze and quantify potential differences in health, resources, care and treatment process after stroke for patients with pre-stroke psychosis or schizophrenia (referred to as pre-stroke psychosis from here), compared to other stroke patients.

## Methods

This study is based on a database including data for Swedish stroke patients identified via ICD-10 codes for acute ischemic stroke (I63^*^) in regional administrative systems and in the Swedish Stroke Register (a national quality registry with a coverage rate of >90% at the time) (18). All stroke cases during 2008-2011 were included from seven regions covering approximately 70% of Sweden's population, including both rural and urban areas as well as Sweden's three major cities. Data extraction was performed in 2013 and the study population was selected to enable complete follow-up. Relevant data from several other research registries were linked on patient level in addition to administrative and disease-specific data; socioeconomic and mortality data from Statistics Sweden, data on municipality services from the National Board of Health and Welfare, and data on filled prescriptions from the Prescribed Drugs Registry, previously shown to be appropriate for scientific research (19). Patients registered in the Swedish Stroke Register received written information and an option to withdraw. All data were pseudonymized and the study was approved by the Regional Ethical Review Board in Stockholm (2013/1541-31/5 and 2016/785-32).

Stroke patients with a history of psychosis diagnosis were identified by the presence of at least one care event related to psychosis according to ICD-10 codes F20-F29. Inclusion in the pre-stroke psychosis subgroup was based on main or secondary diagnosis registered within inpatient or outpatient care during the 2 years preceding the stroke event. Diagnosis (ICD-10), procedure codes, and prescription (ATC) codes were used for the identification of diagnoses, procedures, and filled prescriptions, specifically endarterectomy (PAF20-22), antihypertensive medication (C02-10), and oral anticoagulant (BO1). The Swedish Stroke Register's questionnaires were used for identification of reperfusion treatment, prescriptions at discharge, and for all patient-reported outcomes. Variables from the Swedish Stroke Register were also used for functional status, measured with an approximation of the modified Rankin Scale (mRS) at 3 months and 1 year after the stroke event, in accordance with the method developed by Eriksson et al. (20).

Only patients with ischemic stroke were included in this study (ICD-10 I63^*^). The analysis included comparisons between patients with and without pre-stroke psychosis regarding:

- Health results (survival, functional status, stroke recurrence)
- Treatment interventions and care process (reperfusion, surgical intervention)
- Resource utilization patterns (inpatient and outpatient care, municipality care)
- Secondary pharmacological prevention (filled prescription) patterns

Key indicators were selected to allow for a broad comparison between the two groups, including the most relevant outcome measures for evaluation of stroke care delivery in terms of health, resources, and process. Levels of selected resource indicators before and after stroke were also analyzed (inpatient care level the year before was not adjusted for inpatient care the year preceding the stroke event, as were all other indicators as an approximation for existing comorbidity).

Multivariate regression analysis was performed, and variables for risk adjustment included the baseline characteristics summarized in Table 1, excluding NIHSS at hospital arrival due to a low coverage rate (42%). Hypertension and atrial fibrillation were included for the analysis of stroke recurrence. Regression analyses were performed to address the null hypothesis that there would not be any detectable differences between the psychosis group and the non-psychosis group of stroke patients. Health result indicators were modeled as binary outcomes (logistic regression), as were treatment interventions and filled prescriptions. Resource utilization indicators were treated as count variables (negative binomial regression), with the exception of added home-help service (defined as the difference in home help from the year pre-stroke) which was modeled as a continuous variable (linear regression). Adjustment for age differences was performed with 10-year age groups in the regression analysis. Only 1-year survivors were included in the analyses of resource utilization during the first full-year succeeding the stroke event. Analysis of oral anticoagulants were performed for patients with a history of atrial fibrillation only. Analysis on functional status (approximated mRS) was performed for individuals with all required input data, but we also present the crude results accounting for missing input data and deceased patients. The size of analysis population per indicator is presented in Table S1.

**Table 1:** Baseline characteristics for patients with ischemic stroke, 2008–2011.

	**Pre-stroke psychosis**	**95% CI**	**No psychosis**	**95% CI**
Number of patients (%)	389 (0.84%)		45,961 (99.16%)	
Age in years (mean)	71.1	(69.9; 72.4) (IQR: 19)	76.6	(76.5; 76.7) (IQR: 17)
Sex (% male)	41.4	(36.5; 46.3)	50.3	(49.8; 50.7)
**Highest level of education (distribution, %)**				
- Elementary	49.5		47.7	
- High school	35.7		36.1	
- College/University	14.8		16.2	
**Marital status (distribution, %)**				
- Married	15.7		42.9	
- Never married	37.0		10.6	
- Divorced	27.3		16.4	
- Widowed	20.0		30.1	
Born outside the EU (%)	8.2	(5.5; 11.0)	4.6	(4.4; 4.7)
Living alone (%)	77.5	(73.3; 81.7)	51.0	(50.5; 51.4)
**Living arrangements (distribution, %)**				
- Living at home, no home-help service	41.1		72.3	
- Living at home, with home-help service	27.3		18.7	
- Special housing	31.6		9.0	
ADL-dependency (%)	20.3	(16.2; 24.4)	11.1	(10.8; 11.4)
Prior stroke (−2 years) (%)	8.2	(5.5; 11.0)	6.7	(6.4; 6.9)
Inpatient care year −1 (mean days)	14.5	(10.9; 18.2) (IQR: 16)	5.1	(5.0; 5.3) (IQR: 4)
Atrial fibrillation (%)	24.2	(19.9; 28.4)	30.7	(30.3; 31.1)
Hypertension (%)	53.0	(48.0; 57.9)	62.3	(61.9; 62.7)
**Unconscious at arrival (%)**				
- Conscious	80.7		85.7	
- Indolent	14.3		10.8	
- Unconscious	5.0		3.5	
NIHSS at arrival	4 (50th percentile)	IQR: 6 Min: 0 Max: 37	3 (50th percentile)	IQR: 7 Min: 0 Max: 42

## Results

The proportion of women and the level of inpatient care during the year preceding the stroke event were significantly higher in the pre-stroke psychosis group (Table 1). A higher percentage of patients with a psychosis diagnosis lived in special housing prior to the stroke, and a larger proportion of the psychosis group lived alone and was born outside of the EU. Patients with pre-stroke psychosis showed an almost two-fold proportion of ADL dependency.

**Figure 1:**
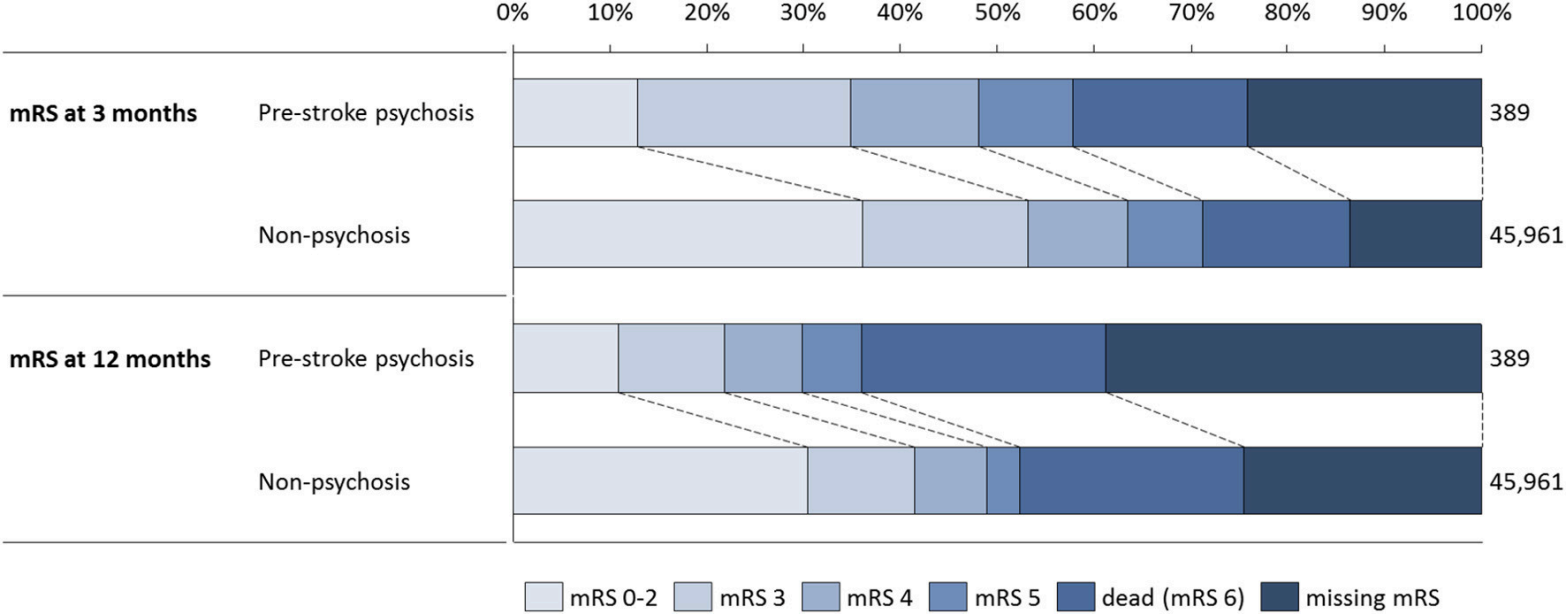
Distribution over approximated mRS categories for the two subgroups, at 3 months and 1 year.

Approximated mRS levels for the two groups at 3 months and 1 year are shown in Figure 1. Functional outcome was notably poorer in the psychosis group, and the crude proportion of patients with mRS 0-2 in the non-psychosis group was equivalent in size to the proportion of patients with either mRS 0-2 or mRS 3 in the psychosis group at 3 months, and equivalent to the proportion of patients in the psychosis group with either mRS 0-2, mRS 3, or mRS 4 at 1 year. At 3 months, input data for approximating mRS (living individuals) were missing for 29.5% of the stroke cases in the psychosis group, and for 15.9% of the non-psychosis group. At 1 year, data for approximating mRS were missing for the majority of patients with a psychosis diagnosis (51.9%), and for 31.9% of the non-psychosis group. The differences in functional status between the subgroups become more pronounced when only patients with complete mRS input data were included.

Only the adjusted results are referred to in the following section if not further specified. One-month and 1-year survival did not differ significantly between the groups as shown in Table 2A, but stroke recurrence was more common in the pre-stroke psychosis group. Patients with a history of psychosis reported significantly lower levels of patient-reported functional status (approximated mRS at 3 months and 1 year).

**Table 2:** Crude and adjusted values (odds ratios/coefficients) for selected indicators between the psychosis and non-psychosis groups.

	**Pre-stroke psychosis**	**Non-psychosis**	**Unadjusted OR (CI 95%)**	**Adjusted OR (CI 95%)**
**A. Health results**				
28-day survival (%)	87.7 (84.4; 90.9)	89.6 (89.3; 89.9)	0.82 (0.61; 1.11)	1.07 (0.71; 1.62)
1-year survival (%)	74.8 (70.5; 79.1)	76.9 (76.6; 77.3)	0.89 (0.71; 1.12)	1.23 (0.90; 1.68)
1-year recurrent stroke (%)	8.6 (5.4; 11.8)	5.9 (5.6; 6.1)	1.50 (0.99; 2.27)	1.57 (1.01; 2.44)
Good 3-months functioning (approximated mRS 0-2, %)	22.2 (16.7; 27.7)	50.6 (50.1; 51.1)	0.28 (0.20; 0.38)	0.44 (0.29; 0.66)
Good 1-year functioning (approximated mRS 0-2, %)	30.0 (22.3; 37.7)	58.1 (57.5; 58.7)	0.31 (0.22; 0.44)	0.40 (0.24; 0.65)
	**Pre-stroke psychosis**	**Non-psychosis**	**Unadjusted OR (CI 95%)**	**Adjusted OR (CI 95%)**
**B. Treatment interventions and care process**				
Reperfusion treatment (thrombolysis or thrombectomy, %)	3.4 (1.6; 5.2)	6.8 (6.5; 7.0)	0.48 (0.28; 0.84)	0.53 (0.29; 0.98)
Endarterectomy within 14 days (%)	0.3 (0.0; 0.8)	1.4 (1.3; 1.5)	0.18 (0.03; 1.31)	^*^
	**Pre-stroke psychosis**	**Non-psychosis**	**Unadjusted coefficient (CI 95%)**	**Adjusted coefficient (CI 95%)**
**C. Resource utilization**				
Initial inpatient stay (days)	12.8 (11.8; 13.9)	13.2 (13.1; 13.3)	−0.03 (−0.12; 0.69)	−0.04 (−0.15; 0.06)
Inpatient care first year (days)	33.7 (28.4; 38.9)	20.9 (20.6; 21.3)	0.48 (0.36; 0.59)	0.32 (0.20; 0.44)
Inpatient care first year excluding at psychiatric unit (days)	25.1 (21.1; 29.2)	20.5 (20.2; 20.8)	0.20 (0.08; 0.32)	0.06 (−0.06; 0.19)
Outpatient care first year (visits)	19.7 (16.9; 22.4)	19.2 (19.0; 19.5)	0.02 (−0.09; 0.13)	−0.01 (−0.12; 0.11)
Added home-help services (hours)	156 (71; 240)	125 (119: 130)	30.8^**^ (−33.0; 94.5)	25.8^**^ (−40.3; 91.9)
	**Pre-stroke psychosis**	**Non-psychosis**	**Unadjusted OR (CI 95%)**	**Adjusted OR (CI 95%)**
**D. Secondary pharmacological prevention**				
Antihypertensive medication (%)	68.7 (63.4; 74.1)	84.1 (83.7; 84.5)	0.42 (0.32; 0.53)	0.55 (0.42; 0.73)
Oral anticoagulant^***^ (%)	40.4 (26.6; 54.2)	55.0 (53.9; 56.1)	0.55 (0.32; 0.97)	0.54 (0.28; 1.03)

*Adjusted values account for differences in case-mix factors as stated in Table 1, excluding NIHSS at hospital arrival, due to the low coverage rate (42%)*.

* *Omitted from regression model due to perfect co-linearity (sample too small)*.

** *Refers to mean number of added hours of home help for the psychosis group (modeled as continuous variable)*.

*** *Proportion of subgroup with diagnosis of atrial fibrillation in the past 2 years*.

The psychosis group had lower levels of reperfusion treatment relative to the non-psychosis group. Degree of enrollment at a specialized stroke unit in direct relation to hospital arrival did not differ between the groups according to the 95% confidence interval (CI) of crude values: 72.5% (67.6; 77.5) in the psychosis group and 75.0% (74.6; 75.5) in the non-psychosis group. The proportion of patients receiving endarterectomy within 14 days also did not differ between the groups. The proportion of patients discharged from acute stroke care to a rehabilitation clinic was 10.5% (95% CI: 7.0; 14.0) in the psychosis group and 12.3% (95% CI: 12.0; 12.7) in the non-psychosis group.

Length of stay after a stroke event did not differ significantly between the groups, as shown in Table 2C. However, the level of inpatient care for the first full year was significantly higher in the pre-stroke psychosis group (this difference was not significant when excluding inpatient care at psychiatric unit). The mean length of stay at a specialized stroke unit (classified according to the Swedish Stroke Register) was 10.4 days (95% CI: 10.2; 10.5) and did not differ between the groups. There was also no difference between the groups in outpatient visits the year after stroke, and the added number of hours of home-help services was not significantly higher in the psychosis group.

Filled prescriptions for antihypertensive medication were significantly less common in the psychosis group, as shown in Table 2D. Differences in dispensation of oral anticoagulants in the subgroup with documented atrial fibrillation was not statistically significant (these levels were however significantly lower for psychosis patients when including the full study population and using atrial fibrillation as a case-mix factor). Prescription levels for antivitamin K at discharge were 7.9% (95% CI: 4.8; 11.0) for the psychosis group and 13.3% (95% CI: 12.9; 13.6) for the non-psychosis group according to the Swedish Stroke Register, looking at the full study population.

**Figure 2:**
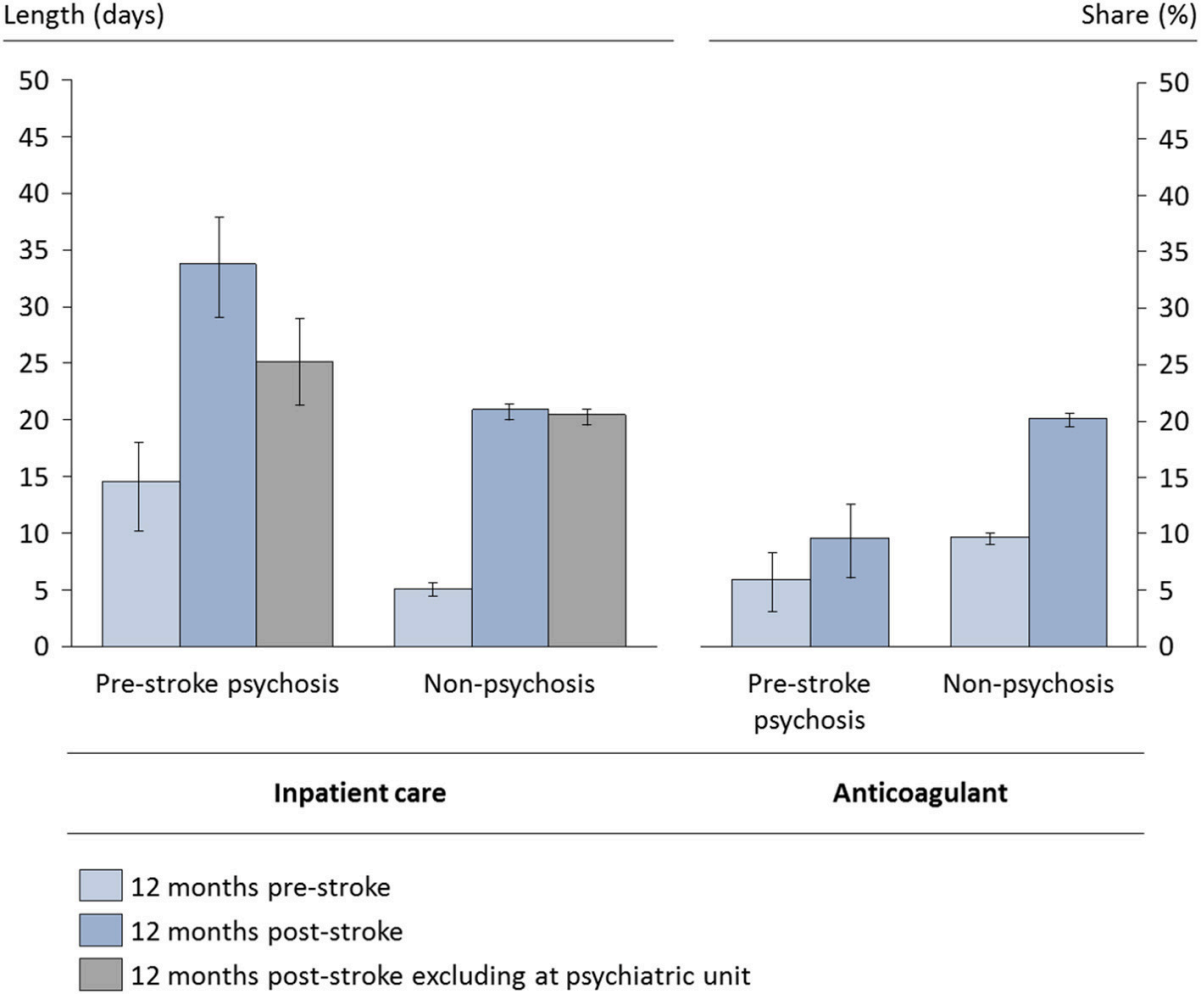
Selected resource indicators before and after stroke. Based on the full study population alive at 1 year. Crude levels, 95% confidence intervals.

The level of inpatient care was significantly higher in the psychosis group the year preceding the stroke event as well as in the succeeding year, while the relative increase in inpatient care during the succeeding year was higher for non-psychosis patients (Figure 2). Levels of filled anticoagulant prescriptions during the second year after stroke were similar to levels during the first year. Both groups showed significantly lower levels of inpatient care during the second year compared to the first year after stroke (data not shown). In addition to history of psychosis, several factors included in Table 1 were significantly associated with the indicators in Table 2, but these findings are outside of the scope of this manuscript.

## Discussion

Individuals with a history of psychosis are vulnerable; we found that pre-stroke psychosis patients were less likely to receive reperfusion treatment, less likely to report good post-stroke functional outcomes, had lower levels of dispensation of drugs for secondary prevention, and had a higher risk of recurrent stroke. These results emphasize the importance of taking mental comorbidity into account during stroke treatment, as it may have a significant impact on stroke care and its results.

We found that acute reperfusion treatment was less utilized in patients with ischemic stroke who had a history of psychosis. A lower likelihood of reperfusion treatment was also found in patients with pre-stroke dementia in a recent analysis of thrombolysis in Swedish patients (21). However, this difference disappeared when adjusting for pre-stroke functional status. In the present study, the difference between subgroups persisted after adjusting for such factors. This could reflect difficulties in identifying stroke symptoms in this patient group by the patient themselves and by health personnel, thus delaying stroke identification and thereby reducing the chance of receiving reperfusion treatment. Such delay might be mitigated via better information regarding early stroke symptoms for this particular group of patients. The pattern observed in this study is in line with a Canadian population-based study which found that patients with a history of psychosis have a much lower chance of receiving guideline-recommended acute treatment for ischemic heart disease (16). We found that despite higher levels of resource utilization (such as inpatient care), health outcomes including functional outcome and stroke recurrence were poorer for these patients, after adjusting for pre-stroke conditions. The levels of filled prescriptions for antihypertensives were also significantly lower, pointing to suboptimal post-stroke treatment and rehabilitation. Poorer secondary prevention has been shown previously in ischemic heart disease (16), and a study from the US reported that the Framingham 10-year risk of coronary heart disease was increased by 79% in patients with schizophrenia (9). One potential driver of poorer cardiovascular health in these patients could be the cardiovascular side effects of antipsychotic drugs (13, 22).

Individuals with mental disorders have been reported to face barriers to medical care for a variety of reasons, including difficulty seeking it and affording it (23). In the Swedish setting, the former reason may be more likely than the latter. More severe comorbidity and lower socioeconomic status are also recurring themes when explaining the difference in access (24, 25), but none of these should be relevant to the results of the present study, as these factors have been adjusted for. Lower levels of adequate procedures have also been explained as related to the fact that cognitive, affective, and social manifestations may complicate the care for patients with psychosis, with specific difficulties related to informed consent and effective rehabilitation (14). These arguments could apply to long-term care including first-year inpatient care and rehabilitation, but not to acute treatment interventions such as reperfusion, or secondary pharmacological prevention prescriptions.

These results are generally aligned with previous research on differences between pre-stroke and non-pre-stroke psychosis patients, with some notable exceptions. Survival was not lower in the psychosis group, as found in a Canadian sample of equivalent size (16). However, Kang et al. (26) found mortality after stroke to be lower in patients with schizophrenia. Both studies adjusted for differences in demographics and comorbidity.

Pre-stroke somatic prerequisites were significantly different between patients with and without a psychosis diagnosis. The proportion of women with pre-stroke psychosis was the inverse of the general psychosis prevalence distribution between sexes, in that women are overrepresented in the stroke population (59%), while men are generally overrepresented in the overall population diagnosed with psychosis (factor 1·4:1 or 58%) (1). Previous data have suggested that somatic comorbidity for patients with psychosis is more common in women than in men (27). This is clearly an important topic for future research. The psychosis subgroup may also have been underdiagnosed with comorbidities such as atrial fibrillation and hypertension, as there is no obvious reason other than a lower age for the healthier levels seen in patients with psychosis (the data in this study indicate that these patients are overall worse off in terms of ADL dependency and need for special housing). The generally higher somatic comorbidity in patients with mental disorders (6, 7) implies an underdiagnosing of cardiovascular risk factors in the psychosis subgroup. It has been shown previously that cardiometabolic risk factors and the risk of cardiovascular disease increase after antipsychotic drug treatment (13, 28).

This study highlights the need for further research on a variety of topics. The reduced number of filled prescriptions found in this study could depend on a lower frequency of prescriptions, a lower compliance in filling the prescriptions, or a combination of both. Patients with pre-stroke psychosis seem to need additional support for enhancing compliance, regardless of the cause. Hippisley-Cox and colleagues found that patients with schizophrenia were less likely to receive statin prescriptions, but no difference was found for anticoagulant prescriptions (29). This could not be addressed in the present study, as data extracts from the Prescribed Drug Registry cover only filled prescriptions. Prescription levels at discharge were even lower than levels of filled prescriptions for the first full year after stroke according to data from the Swedish Stroke Register, implying that patients may rely on prescriptions from sources other than the stroke unit managing the acute phase of treatment. Non-antivitamin K anticoagulants were not used as frequently during the study period (2008–2011) as at the time of the publication of the results.

This study is one of the first to focus on patients with pre-stroke psychosis in a health system characterized by universal, tax-subsidized access to healthcare. It aimed at performing a holistic assessment of the health results, treatment interventions and care process, resource utilization, and secondary pharmacological prevention for these patients. It was based on observational registry data covering most of Sweden's stroke patients with pre-stroke psychosis for the time interval studied. The study design did not allow for control of systematic bias in registering, but its patient-level linkage of data from several national registries enabled case-mix adjustments for a broad range of patient characteristics, clinical, and socioeconomic factors. It would have been of interest to prolong the time horizon for indicators studied as well as to enlarge the study population, but this was not possible with the available data.

In conclusion, patients with pre-stroke psychosis were found to have a lower degree of reperfusion treatment, a poorer outcome, and poorer utilization of secondary pharmacological prevention. Therefore, the initial null hypothesis that there would not be any detectable differences between the groups is rejected. These results emphasize the importance of considering mental comorbidity during stroke treatment and when evaluating or comparing health results, resource utilization, and care process for stroke patients, as mental comorbidities such as psychosis and schizophrenia may have a significant impact on health regained and care resources utilized the year preceding and the year succeeding the stroke event. These differences are likely not limited to the first year after stroke.

## Author contributions

CW, KS, and MvE conceived the idea of the study. CW and IL performed data management and statistical analyses. CW interpreted the analyses and drafted the manuscript. All authors planned the data collection, discussed and jointly decided upon factors for multivariate case-mix adjustments, and critically revised the manuscript.

### Conflict of interest statement

CW and IL are employed by Ivbar Institute, a research company specialized in healthcare governance and analysis of healthcare data. IL also holds stock in the company. The remaining authors declare that the research was conducted in the absence of any commercial or financial relationships that could be construed as a potential conflict of interest.
