# Supplementary material for: The Association of Pre-stroke Psychosis and Post-stroke Levels of Health, Resource Utilization, and Care Process: A Register-Based Study
Source: Front Neurol. 2018 Dec 3;9:1042. doi: 10.3389/fneur.2018.01042 (PMC6287012; doi:10.3389/fneur.2018.01042)
Supplement: Supplementary file 1 [file Table_1.docx]

**Table S1**. Size of analysis population by indicator.

| Indicator | Total | Psychosis group | Non-psychosis group |
| --- | --- | --- | --- |
| 28-day survival | 43,162 | 334 | 42,828 |
| 1-year survival | 43,162 | 334 | 42,828 |
| 1-year recurrent stroke | 33,728 | 258 | 33,470 |
| Good 3-months functioning (approximated mRS 0-2) | 31,415 | 200 | 31,215 |
| Good 1-year functioning (approximated mRS 0-2) | 23,227 | 123 | 23,227 |
| Reperfusion treatment  (thrombolysis or thrombectomy) | 43,438 | 343 | 43,095 |
| Endarterectomy within 14 days | 43,778 | 345 | 43,433 |
| Initial inpatient stay | 43,162 | 334 | 42,828 |
| Inpatient care first year | 33,728 | 258 | 33,470 |
| Inpatient care first year excluding at psychiatric unit | 33,728 | 258 | 33,470 |
| Outpatient care first year | 33,728 | 258 | 33,470 |
| Added home-help services | 31,257 | 215 | 31,042 |
| Antihypertensive medication | 33,950 | 264 | 33,686 |
| Oral anticoagulant | 8,506 | 52 | 8,454 |
